# Implications for safety: *ASFV-G-ΔI177L* vaccine compromises health and semen quality in adult breeding boars

**DOI:** 10.3389/fmicb.2026.1823118

**Published:** 2026-04-29

**Authors:** Virginia Friedrichs, Sten Calvelage, Theresa Holzum, Elisenda Viaplana, Alicia Urniza, Monica Balasch, Martin Beer, Sandra Blome, Alexander Schäfer

**Affiliations:** 1 Institute for Diagnostic Virology, Friedrich-Loeffler-Institut, Greifswald–Insel Riems, Germany; 2 Zoetis Manufacturing and Research Spain, Finca la Riba, L’Hostalnou de Bianya, Girona, Spain

**Keywords:** African swine fever virus, breeding boar, domestic pig, genetic stability, live attenuated vaccine, semen quality, vaccine safety

## Abstract

African swine fever poses an ongoing threat to Eurasian wild pigs and the associated global swine production, with no internationally licensed vaccine available. Live attenuated vaccines like *ASFV-G-ΔI177L* demonstrated promising safety and protection in young domestic pigs, but comprehensive safety evaluations in breeding animals are necessary for regulatory licensing in the EU. This study investigated the safety of intramuscular immunization with *ASFV-G-ΔI177L* in adult breeding boars (*Sus scrofa domesticus*). Eight healthy boars were intramuscularly vaccinated and monitored for clinical manifestation, viral genome and infectious virus in blood, oral fluid, semen, and tissues. Furthermore, ASFV-specific antibodies and effects on semen quality were assessed over an observation period of 28 days. Clinical signs appeared in all boars at 6 days post vaccination (dpv), including high fever, lethargy, and anorexia in most animals by 8 dpv. Four animals developed severe disease and were euthanized at humane endpoints or succumbed between 11 and 15 dpv, while four animals recovered. Viral genome was detected in blood, oral fluid, and semen of all animals, with higher loads in succumbed individuals. Infectious *ASFV-G-ΔI177L* was found in reproductive tissues, spleens, salivary glands, and semen samples from acutely affected boars and one recovered boar. All boars developed ASFV-targeting antibodies, but titers remained low. Semen quality was significantly reduced after vaccination and failed to recover in surviving boars by 28 dpv. These findings raise concerns regarding vaccine safety in reproductively active boars and highlight the risk of virus transmission through semen.

## 1 Introduction

African swine fever (ASF) is an often-fatal disease of domestic (*Sus scrofa domesticus*) and Eurasian wild suids, caused by African swine fever virus (ASFV), an enveloped, double-stranded DNA virus of the *Asfarviridae* family. The disease typically results in high morbidity and lethality rates, accompanied by severe economic losses and threats to the global pork production (Nguyen-Thi et al., 2021; You et al., 2021). Clinical manifestations range from peracute death to chronic forms. While clinical signs may vary between strains, they usually include fever, lethargy, anorexia, and hemorrhagic lesions. ASFV primarily targets monocytes and macrophages, which infiltrate tissues and mediate viral spread (Blome et al., 2013; Salguero, 2020; Ruedas-Torres et al., 2024).

The absence of antiviral treatments and globally licensed vaccines necessitates strict biosecurity, early diagnosis, and culling of infected animals as core control measures. In recent years, considerable effort has produced a wide range of promising vaccine candidates, reflecting the whole spectrum of vaccine preparations: from inactivated vaccines (Pikalo et al., 2022), to vector vaccines (Ravilov et al., 2022), subunit vaccines (Ruiz-Gonzalvo et al., 1996; Goatley et al., 2020), mRNA vaccines (Hu et al., 2025; Yuan et al., 2025), and live attenuated vaccines (LAV) (Liu et al., 2021; Fan et al., 2024; Ntakiyisumba et al., 2025). However, most candidate vaccines have failed in further studies, either due to insufficient safety or inadequate protection against challenge infection as a result of the limited understanding of immunity against ASFV and immunogenicity of the viral proteome (Karger et al., 2019; Schäfer et al., 2022). The vaccines that typically induce the most robust protection against ASFV, and are thus the most promising yet, are LAVs (Urbano and Ferreira, 2022; Fan et al., 2024). Over the last years, a multitude of LAV candidates have been proposed (Fan et al., 2024). However, few have successfully proven their safety, stability, and efficacy in follow-up studies under laboratory conditions. Among them is the LAV candidate *ASFV-G-ΔI177L*, which was proposed by Borca et al. (2020). *ASFV-G-ΔI177L* was analyzed in subsequent studies and was found to be an effective and safe vaccine (Borca et al., 2021; Attreed et al., 2022; Tran et al., 2022a,b; Borca et al., 2023), eliciting a robust T cell memory response (Attreed et al., 2022). However, a necessity for the licensing process is that candidate LAVs must adhere to stringent safety and efficacy requirements before approval.

To obtain licenses for the European market, several safety and efficacy studies are mandated by regulatory authorities. European Union Regulation (EU) 2019/6, the European Pharmacopeia, the WOAH Terrestrial Manual, and associated guidelines specify that ASFV vaccine safety studies should involve assessment in target species under controlled conditions, including different doses and repeated administration, monitoring of clinical signs, transmission potential, viremia, tissue dissemination, and genetic stability upon forced passaging. Key safety endpoints include absence of severe ASF-related clinical signs, minimal horizontal transmission, lack of significant fever, and stability in reversion to virulence testing (Tran et al., 2022b). Special emphasis is placed on monitoring vaccinated breeding animals’ reproductive performance, as previous studies suggest live ASFV vaccine strains can impact fetal and neonatal outcomes and cause residual pathogenicity (Lee et al., 2025; van den Born et al., 2025).

In this study, we evaluated the safety of intramuscular immunization with the LAV candidate *ASFV-G-ΔI177L* in adult breeding boars (*Sus scrofa domesticus*). The trial was conducted in accordance with VICH (International Cooperation on Harmonisation of Technical Requirements for Registration of Veterinary Medicinal Products) guideline 44, Target Animal Safety for Veterinary Live and Inactivated Vaccines (reference number EMEA/CVMP/VICH/359665/2005). Clinical progression, virological parameters, ASFV-specific antibodies, and semen quality were assessed following current regulatory standards for veterinary vaccine testing. This work addresses a key knowledge gap regarding ASFV vaccine safety in boars, with important implications for both disease control and commercial swine breeding.

## 2 Materials and methods

### 2.1 Animal husbandry

Prior to obtaining the animals, the presented study was approved by the regional competent authority for animal welfare, the Landesamt für Landwirtschaft und Fischerei Mecklenburg-Vorpommern (LALLF-MV) and is listed under reference number 7221.3–1-008/25 approved on March 12, 2025. The trial was conducted in accordance with current German animal welfare law. The boars were kept in the high containment facilities at the Friedrich-Loeffler-Institut (FLI) Riems. Two boars each were kept in a total of four large stables (24 m^2^ each), each in an individual pen. The boars were allowed visual contact, but physical contact was avoided to prevent hierarchical fighting. They underwent a two-week acclimatization, during which they were handled daily to familiarize them with personnel and reduce stress during sampling.

### 2.2 Study design and sampling

**Figure 1:**
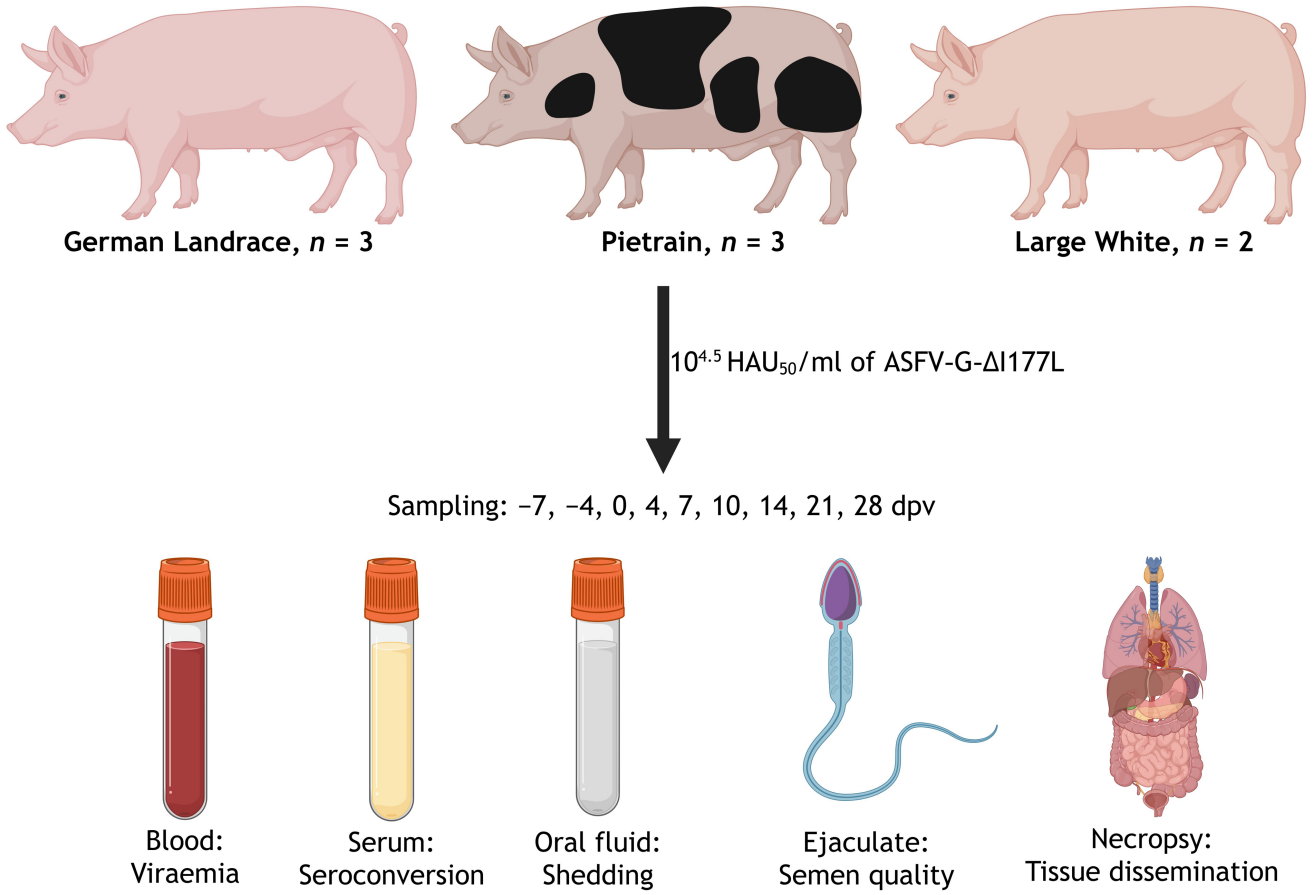
Illustration of the study design. Eight adult breeding boars were intramuscularly immunized with the LAV *ASFV-G-ΔI177L* (1 mL per individual with 10^4.5^ HAU_50_/mL). The animals were sampled −7, −4, 0, 4, 7, 10, 14, 21, and 28 dpv. On each sampling day, EDTA-blood was taken for viremia assessment, serum to check for ASFV-targeting antibodies, oral fluid for shedding evaluation, and the ejaculate to analyze the semen quality. During necropsy, organs were sampled to allow evaluation of vaccine virus dissemination. Generated with BioRender.

Eight mature breeding boars (*Sus scrofa domesticus*) were included in the trial: three German Landrace, two Large White, and three Pietrain boars, sourced from a commercial boar stud in Germany (BHZP = Bundes Hybrid Zucht Programm) with an extensive, regular health monitoring for various pig pathogens. Prior to the transfer, all animals tested negative in qPCR (ASFV genome) and serology (ASFV-specific antibodies detected by immunoperoxide test (IPT)). Besides ASFV, all animals were assessed by direct testing and confirmed free of: Classical swine fever virus, Suid alphaherpesvirus 1 (i.e., Aujeszky’s disease), Porcine reproductive and respiratory syndrome virus, *Actinobacillus pleuropneumoniae*, *Mycoplasma hyopneumoniae*, endo- and ectoparasites (e.g., *Ascaris* spp. and mites), *Brachyspira hyodysenteriae*, and *Salmonella*. Ages ranged between 9 and 21 months and all were trained to mount a dummy. As shown in Figure 1, semen, blood, and serum were collected from all boars at −7, −4, and 0 dpv to establish reference values for each individual. Immediately after sample collection at 0 dpv, all boars were vaccinated intramuscularly with 10^4.5^ HAU_50_/mL of the experimental vaccine *ASFV-G-ΔI177L* (1 mL per individual). The vaccine was produced at industry-grade standard and provided by Zoetis. Since the present study was designed as a safety study in accordance with VICH guideline 44, the intramuscular route was chosen. It is well established that the intramuscular route is more likely to induce adverse vaccination effects compared to the oral route by Xu et al., (2024). During the observation period of 28 days, clinical score and rectal temperatures were recorded daily. Additionally, the semen, blood, and serum samples were collected from all boars 4, 7, 10, 14, 21, and 28 dpv. Semen samples were subjected to comprehensive quality control procedures (see 2.3), as well as qPCR and virus isolation to determine presence of ASFV genome and infectious viral particles. Blood samples were assessed for viraemia after vaccination, whereas serum samples were used to monitor the presence of ASFV-specific antibodies. Blood and serum samples were obtained from the saphenous (*Vena saphena*) or caudal (*V. caudalis mediana*) vein during semen collection. Furthermore, each boar was offered a cotton rope on each sampling day to allow collection of individual oral fluid samples. Oral fluid was collected by placing the rope in a 50 mL centrifugation tube on top of three closed 200 μL tubes and centrifugation at 2,000 × *g*. Oral fluid was subjected to qPCR to enable analysis of vaccine virus shedding.

As this trial was a safety study without highly virulent challenge, a moderate HEP of 10 cumulative points was chosen. The clinical score was determined according to an adapted version of Mittelholzer et al. (2000), including the slight modification of introducing a 4-point scoring system for each parameter instead of the published 3-point scoring system. Upon reaching the HEP or at the end of the observation period 28 dpv, the boars were subjected to necropsy. Each individual was sedated using a combination of 1 mg/kg azaperone (Stresnil, Elanco) and 2.2 mg/kg ketamine (Ketamin 10%, cp-pharma). Euthanasia was conducted by intraventricular injection of pentobarbital sodium (Release, WDT). After euthanasia, gross pathological scores were documented during the necropsy. Additionally, the following organs were sampled and subjected to qPCR and some to virus isolation: tonsil, mandibular lymph node (mLN), salivary gland, lung, liver, gastrohepatic lymph node (ghLN), epididymis, testis, bulbourethral gland, vesicular gland, and prostate. To obtain homogenates for subsequent analysis, an organ piece (weighed, ~200 mg) was placed in a 2 mL centrifugation tube with 1 mL PBS and a 5 mm metal bead, then subjected to homogenization at 30 Hz for 3 min using a tissue lyzer (TissueLyzer II, Qiagen). Furthermore, blood and serum were collected from sedated animals prior to euthanasia.

### 2.3 Semen quality assessment

Semen was collected from each boar using clean gloves and a method, where the ejaculate is directly collected from the penis into a thermal mug without contact to the glove. The thermal mug was fitted with a specialized semen collection bag equipped with inline filters to prevent contamination with bulbourethral secretions (Minitube). Immediately after collection, the whole ejaculate was diluted with Beltsville TS semen extender (Minitube), pre-warmed to 37 °C to maintain spermatozoa viability. Semen quality was assessed during the trial by analyzing the following parameters: (a) total semen volume prior to dilution in mL, (b) spermatozoa count per mL and percent of motile and viable spermatozoa, (c) percent of morphologically abnormal spermatozoa, and (d) presence and count of infiltrating cells, if applicable. Morphological abnormalities included double heads, absence of tail, tail loops caused by proximal cytoplasmic droplets, and cytoplasmic droplets located at the head or tail. Semen quality control followed BHZP protocols, and only parameters not requiring specialized equipment were evaluated during the trial. Aliquots of each semen sample were subjected to qPCR, virus isolation, and storage at −80 °C for later use.

### 2.4 Hemadsorption test and virus isolation

The HATs were performed both to determine the infectious titer of the experimental ASFV vaccine and to detect infectious ASFV particles in semen and organ samples. Initially, peripheral blood mononuclear cells (PBMCs) were isolated from EDTA-blood of a healthy donor pig kept at the FLI quarantine facility. The EDTA-blood was mixed 1:10 with a 10% Hanks dextran solution (Sigma-Aldrich) and incubated for 90 min at room temperature. The PBMC-rich supernatant was collected and washed twice with PBS containing 2.5 mM EDTA to prevent cell aggregation. The fraction containing red blood cells (RBCs) was diluted 1:10 with PBS and kept at 4 °C for later use. The PBMCs were seeded into 96-well Primaria plates (Corning) at a density of 5 × 10^5^ cells/well in Dulbecco′s Modified Eagle′s Medium (DMEM, supplemented with 10% fetal calf serum and 0.01% Penicillin/Streptomycin, Gibco). Plates were incubated at 37 °C with 5% CO₂ for 24 h, after which recombinant porcine colony-stimulating factor 2 (CSF2; 2 ng/mL, Biomol) was added. Following another 24 h incubation, vaccine virus was titrated (a) prior to inoculation of the boars and (b) post inoculation to double-verify the vaccine titer of 10^4.5^ HAU_50_/mL. After 24 h of viral inoculation, donor-specific erythrocytes were added at a ratio of 1:40 to visualize rosette formation at infected monocytes/macrophages. Rosette formation was evaluated after 24 h and 48 h.

Virus isolation was performed on organ samples (salivary gland, spleen, bulbourethral gland, vesicular gland, prostate, testis, and epididymis) and on semen collected 10, 14, 21, and 28 dpv. For initial blind passage, PBMCs were seeded into 24-well plates at 2.5 × 10^6^ cells/well and differentiated with CSF2 as above. Of tissue homogenate or semen, 100 μL were added in duplicate, incubated for 72 h, and then frozen at −80 °C to lyse remaining cells. A HAT was subsequently performed on supernatants, with each blind passage replicate (*n* = 2) tested in quadruplicate (*n* = 4) in the assay. Results were scored as negative (all wells negative), weak-positive (up to 4 wells positive), positive (4–8 wells positive), and strong positive (4–8 wells positive, high rosette counts).

### 2.5 DNA extraction and qPCR

Nucleic acids were extracted from 100 μL EDTA-blood, tissue homogenate, or oral fluid, and 80 μL semen using the NucleoMag VET Kit (Macherey-Nagel) on a KingFisher Flex 96 system (Thermo Fisher Scientific) according to the manufacturer’s instructions. ASFV genome-negative serum served as an extraction control.

All qPCR reactions were performed using the virotype ASFV 2.0 PCR kit (Indical), including the manufacturer’s internal amplification control to monitor inhibition. All semen samples were extracted twice to maximize detection sensitivity. In addition to the virotype ASFV 2.0 PCR kit, the VetMAX African Swine Fever Virus Detection Kit (Thermo Fisher Scientific), previously shown to be highly sensitive for ASFV detection in boar semen (Friedrichs et al., 2024), was used to confirm results from the first extraction. Internal control DNA provided in the kit was added to the second extraction. To enable calculation of ASFV genome copies in each sample, a standard was included in each run, with known ASFV genome copy numbers ranging from 10^0^ to 10^7^ copies per mL extracted DNA. All qPCR reactions were run on a Bio-Rad C1000 thermal cycler equipped with the CFX96 Real-Time System (Bio-Rad).

### 2.6 Serology

All serum samples were tested for ASFV-specific antibodies using two commercial ELISA kits: (a) ID Screen ASF Competition (ID.vet), detecting antibodies against ASFV-p32, and (b) Ingezim PPA COMPAC (Gold Standard Diagnostics), detecting anti-ASFV-p72 antibodies. Assays were performed according to manufacturers’ protocols. In parallel, all serum samples were tested by IPT, which served as the reference serological assay due to its superior sensitivity. IPT was done following the European Union Reference Laboratory for ASF (EURL-ASF) protocol for the detection of antibodies against ASFV by IPT (SOP/CISA/ASF/IPT/1, 2018) with slight modifications regarding the strain and cell type. Samples positive in IPT were titrated in twofold dilutions, starting at 1:40, to determine semi-quantitative antibody titers of each positive serum sample.

### 2.7 Sequencing

To monitor the genetic stability of the utilized *ASFV-G-ΔI177L* vaccine strain candidate during the course of the experiment, samples of succumbed and surviving animals as well as a batch of the administered inoculum were processed for 2nd and 3rd generation sequencing. Therefore, DNA was extracted from 200 μL blood and macrophage cultures by adding 180 μL VL-1 buffer for lysis and 20 μL proteinase K from the NucleoMag VET Kit (Macherey-Nagel) and the subsequent processing of the 400 μL lysate with the RNAdvance Tissue Kit (Beckmann Coulter) on a KingFisher Flex 96 system (Thermo Fisher Scientific) following manufactures instructions. Quantification and quality check of the obtained DNA was performed on an NanoPhotometer N60 (Implen) followed by an optional clean-up step with AMPure XP Beads (Beckmann Coulter) to remove residual contaminations or concentrate DNA if necessary.

Next-generation sequencing was conducted on an Illumina NextSeq 2000 (Illumina) for short-read sequencing and on a PromethION P2 solo for long-read nanopore sequencing (Oxford Nanopore Sequencing). For the preparation of Illumina-compatible libraries, 350 ng (spleen sample) or 19.5 ng (inoculum) DNA were used as input for DNA fragmentation on an M220 Focused-ultrasonicator (Covaris). The sheared DNA was concentrated with AMPure XP Beads and further processed with the NEBNext Ultra II DNA Library Prep Kit for Illumina (NEB #E7645L) and NEBNext Multiplex Oligos for Illumina (Unique Dual Index UMI Adaptors DNA Set 4; NEB #E7878S) following the manufactures instructions for library construction. After a size-selection step, libraries were quantified using the universal KAPA Library Quantification Kit for Illumina (Roche) and quality checked by electrophoresis performed on a 2,100 Bioanalyzer system and High Sensitivity DNA chip and reagent kits (Agilent). Final libraries were pooled and sequenced on a NextSeq 1000/2000 P2 XLEAP-SBS Reagent Kit (600 Cycles; Illumina) according to manufactures instructions.

Library preparation for long-read sequencing was conducted using the Rapid Barcoding Kit (SQK-RBK114; Oxford Nanopore Technologies) with DNA inputs ranging between 68 and 200 ng per sample. The pooled libraires were sequenced on an R10.4.1 PromethION Flow Cell (FLO-PRO114M; Oxford Nanopore Technologies) in super-accurate basecalling mode (400 bps).

### 2.8 Sequencing data analysis

Illumina datasets were mapped against a previously generated *ASFV-G-ΔI177L* reference (lib06797) using Newbler 3.0 (Roche) with default parameters including adapter and quality trimming. For live-basecalling of long-read raw data, the Dorado basecaller was used to generate the corresponding FASTQ files (v. 7.8.3 for lib07260-63 and 7.9.8 for lib07385-88) with enabled barcode trimming. Quality trimming of long-read data was achieved by BBDuk (v.1.0) utilizing additional parameters (qtrim = rl, trimq = 6, minlength = 50, ordered = t, qin = 33) followed by the mapping of trimmed datasets against the *ASFV-G-ΔI177L* reference with Minimap2 (v. 2.29, preset parameter: -x map-ont). All alignments were manually inspected in Geneious Prime (v. 2025.1.3) and corrected if necessary.

### 2.9 Statistical analyses

Data analyses and visualization were carried out in GraphPad Prism 10 (GraphPad Software Inc.). Normality was confirmed by Shapiro–Wilk test prior to further analyses. To evaluate the significance of changes in semen quality parameters, a Two-way ANOVA with Tukey post-test between grouped pre- and post-immunization samples was performed. Correlation analyses were done by simple linear regression. Statistically significant differences were defined as *p* < 0.05.

## 3 Results

### 3.1 Intramuscular immunization with *ASFV-G-ΔI177L* is not safe for adult breeding boars

**Figure 2:**
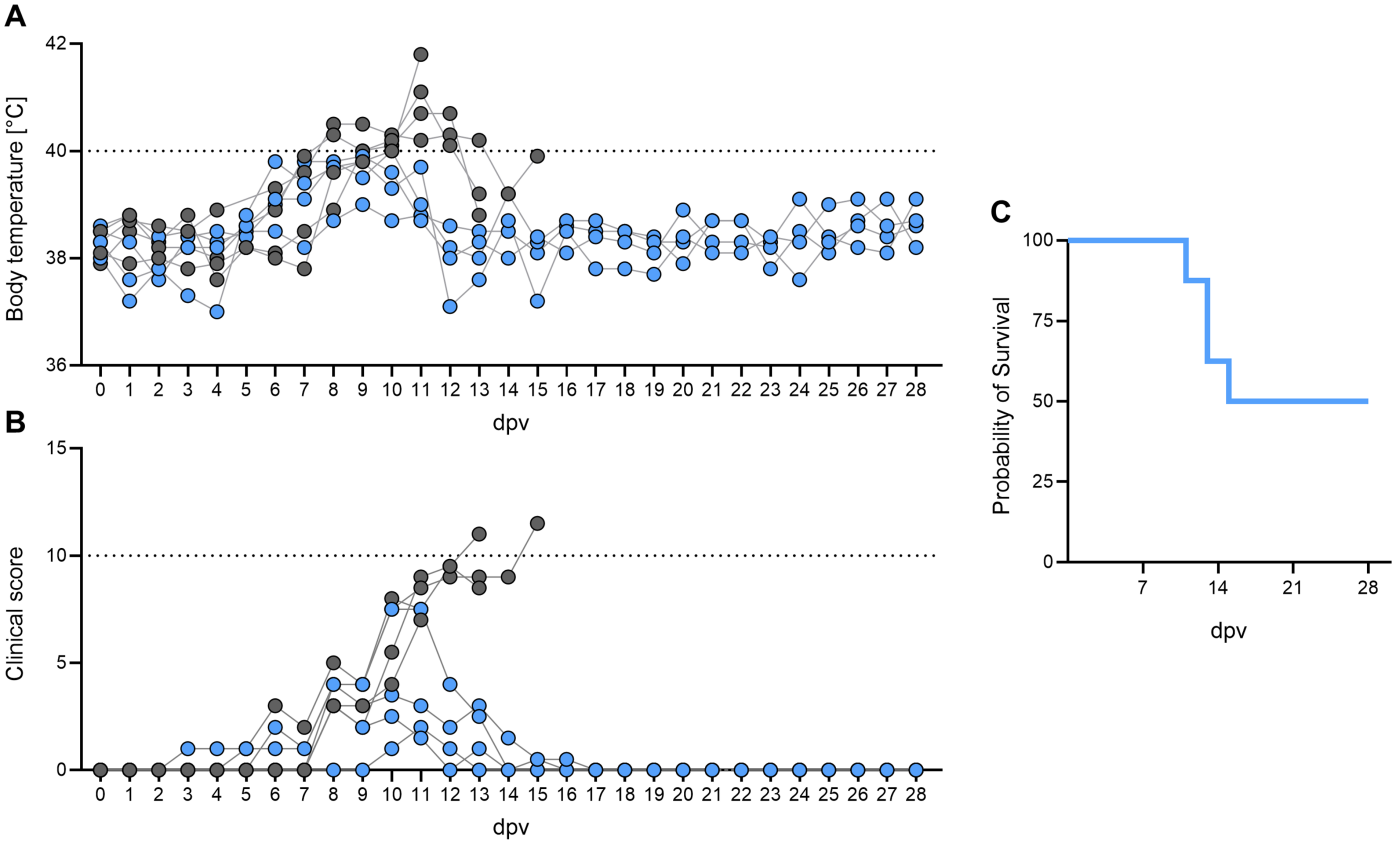
Individual **(A)** rectal temperatures and **(B)** clinical signs, and **(C)** survival in adult breeding boars (*n* = 8) after intramuscular immunization with 1 mL of 10^4.5^ HAU_50_/mL *ASFV-G-ΔI177L*. Animals that succumbed after immunization or reached the HEP are depicted as grey circles. Dotted lines indicate fever threshold (40.0 °C) and the HEP.

Eight healthy, naïve boars were intramuscularly immunized with 1 mL of 10^4.5^ HAU_50_/mL *ASFV-G-ΔI177L*. The first minor, unspecific clinical signs were observed around 6 days post vaccination (dpv), which increased 8 dpv when most animals developed fever and presented with lethargy and anorexia (Figures 2A,B). Four boars had significant clinical signs, mainly fever up to 41.8 °C and associated signs, e.g., lethargy and anorexia. No ASF-specific clinical signs like dermal hemorrhages or cyanosis were observed. Ultimately, these four animals did not recover and succumbed after immunization or were euthanized upon reaching the humane endpoint (HEP) 11–15 dpv (Figure 2C). At necropsy, only mild ASF-specific pathological alterations were detected, comprising sporadic hemorrhagic lymph nodes and ascites in individual boars. Petechial hemorrhages, which are characteristic of acute ASFV infection, were not observed in any case. All non-survivors exhibited pronounced weight loss, and two additionally presented with testicular edema. In the majority of animals that succumbed following immunization, secondary pathological conditions were identified, including bacterial infections (phlegmon with subsequent septicemia) and gastric ulceration. One boar died from acute circulatory failure at a recorded body temperature of 41.8 °C.

The remaining four boars recovered, with fever and clinical signs subsiding 17 dpv at the latest. Sampling in these animals continued until the end of the observation period 28 dpv. Upon necropsy, two of the surviving boars were found to have perihepatitis, one of them also showed signs of pericarditis. The two other boars did not show any pathological findings.

### 3.2 *ASFV-G-ΔI177L* genome is found in blood, oral fluid, and semen of vaccinated boars

**Figure 3:**
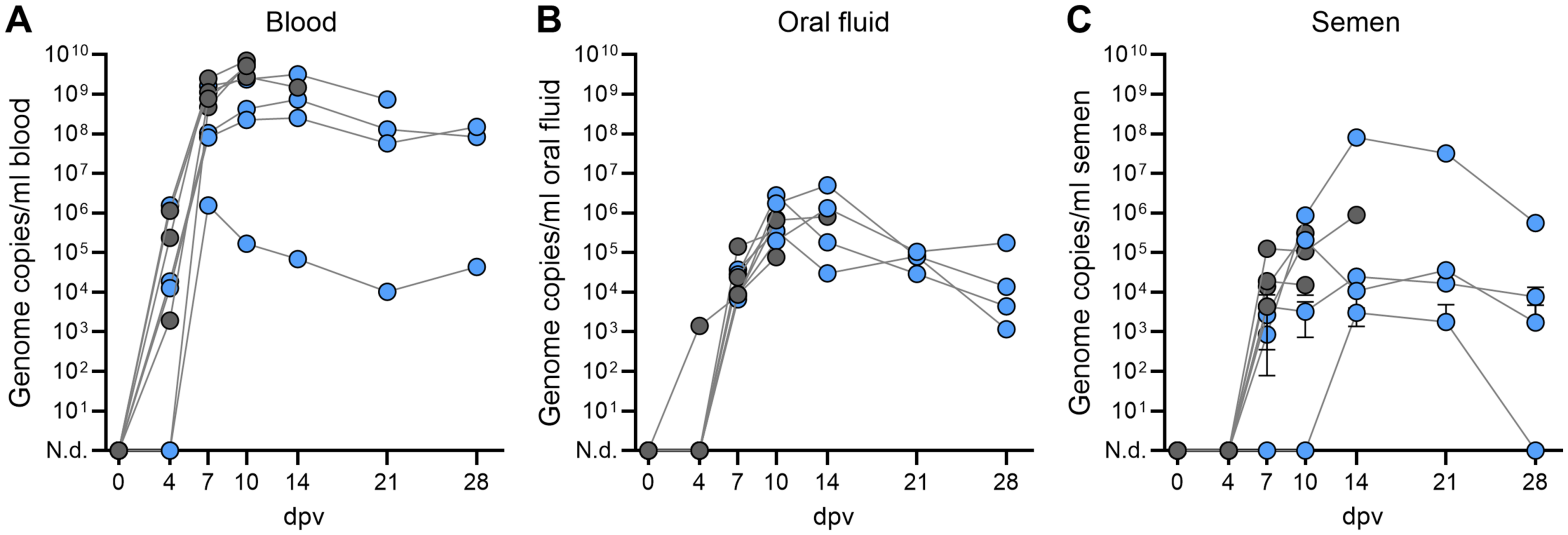
Detection of *ASFV-G-ΔI177L* genome copies in **(A)** blood, **(B)** oral fluid, and **(C)** semen in breeding boars (*n* = 8) after intramuscular immunization with 1 mL of 10^4.5^ HAU_50_/mL *ASFV-G-ΔI177L*. Animals that succumbed after immunization or reached the HEP are depicted as grey circles. A blood sample of boar #7 could not be obtained 28 dpv. N.d., not detected.

Genome copy numbers of *ASFV-G-ΔI177L* in blood, oral fluid, and semen were assessed as a marker for viral dissemination in the animals (Figure 3). *ASFV-G-ΔI177L* genome in the blood was found in most animals 4 dpv and all animals 7 dpv. Genome copy numbers remained relatively stable throughout the observation period (10^5^-10^9^/mL in surviving boars). Boars that eventually did not survive had 1–2 log-fold higher genome copy numbers in the blood than recovered boars (Figure 3A). As evaluation of vaccine virus shedding, oral fluid was tested. Detection of *ASFV-G-ΔI177L* genome in blood preceded detection in oral fluid of the boars, since only one boar was positive in oral fluid 4 dpv. All animals were positive 7 dpv (Figure 3B). Although genome copy numbers were considerably lower in oral fluids than in blood, numbers remained relatively stable throughout the study, with no differences between survivors and non-survivors. In the semen, *ASFV-G-ΔI177L* genome was found in all boars 7 dpv except one (#2), which was positive in later semen samples, 14 and 21 dpv. The same boar displayed only low levels of *ASFV-G-ΔI177L* genome in the semen and was already negative 28 dpv. The other boars had *ASFV-G-ΔI177L* genome copy numbers comparable to those in the oral fluids and remained positive until reaching the respective HEP or the end of the observation period (Figure 3C).

### 3.3 *ASFV-G-ΔI177L* genome and infectious virus are found in various tissues and semen of vaccinated boars

**Figure 4:**
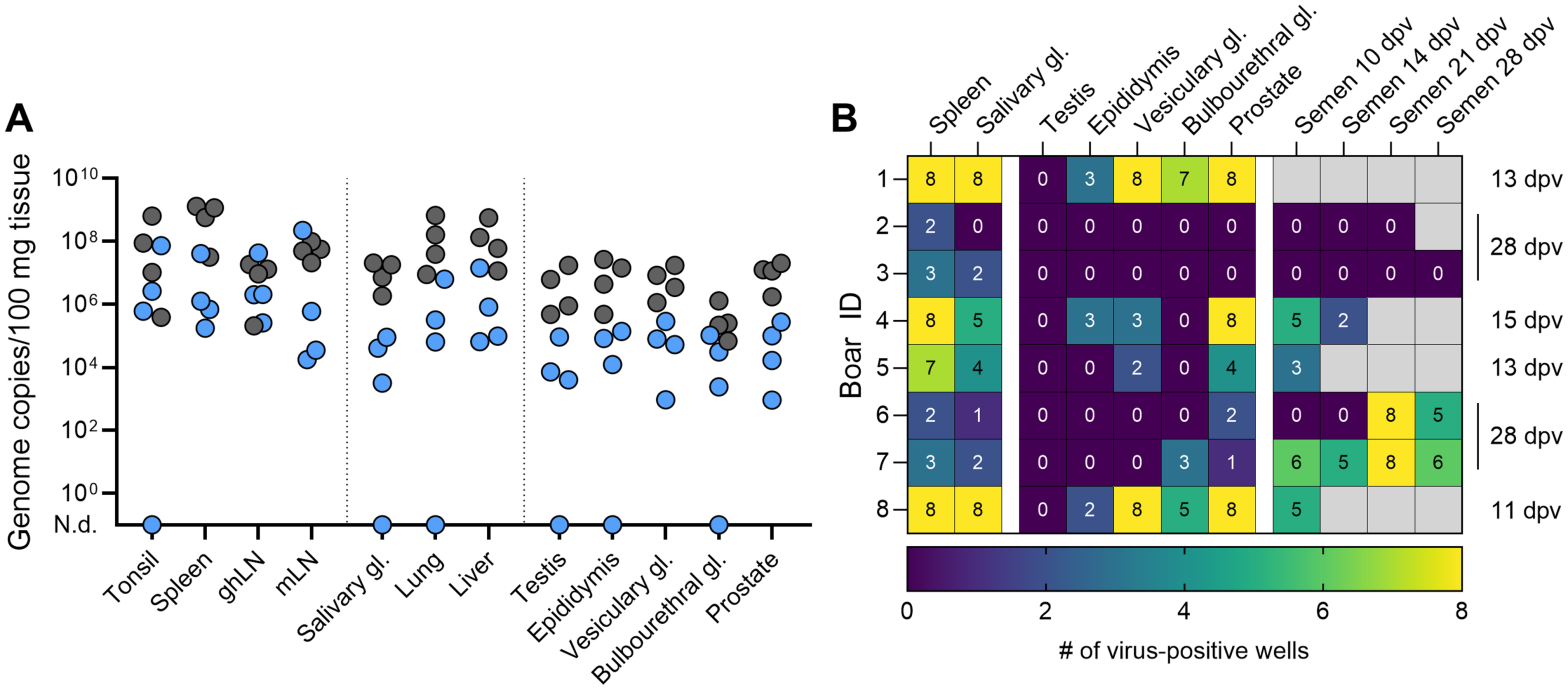
Detection of **(A)** *ASFV-G-ΔI177L* genome copies in lymphoid, non-lymphoid, and reproductive tissues by qPCR, and **(B)** infectious *ASFV-G-ΔI177L* virus by hemadsorption test in the indicated tissues of breeding boars (*n* = 8) after intramuscular immunization with 1 mL of 10^4.5^ HAU_50_/mL *ASFV-G-ΔI177L*. Samples were taken during necropsies either when euthanized at 11–15 dpv or at the end of the trial (28 dpv). Animals that succumbed after immunization or reached the HEP are depicted as grey circles in **(A)**. Numbers in **(B)** indicate the number of virus-positive wells in the HAT, grey squares indicate non-available samples. N.d., not detected; ghLN, gastro-hepatic lymph node; mLN, mandibular lymph node; gl., gland.

During necropsy, several lymphoid (tonsil, spleen, gastrohepatic and mandibular lymph nodes) and non-lymphoid (salivary gland, liver, lung) tissues, as well as samples from reproductive organs (testes, epididymis, vesicular gland, bulbourethral gland, prostate) were taken and investigated for the presence of *ASFV-G-ΔI177L* genome copies. Only one of the surviving boars was negative in some tissues (#2 in tonsil, salivary gland, lung, testis, bulbourethral gland) 28 dpv, while *ASFV-G-ΔI177L* genome was found in all sampled tissues of all other animals (Figure 4A). Acutely deceased boars generally had higher levels of *ASFV-G-ΔI177L* genome in their tissues compared to recovered animals, especially in spleens and reproductive organs. Samples from spleens and salivary glands, all reproductive tissues, and semen samples taken 10, 14, 21, and 28 dpv were investigated in a hemadsorption test (HAT, Figure 4B) to assess whether infectious virus was present in the tissues. Most tissues taken from acutely euthanized boars were highly positive for infectious virus (Figure 4B). In contrast, while there was still some infectious virus detectable in spleens or salivary glands of all boars, the reproductive tissues and semen samples were positive in only two of the recovered boars. There was no infectious virus in any testes.

### 3.4 *ASFV-G-ΔI177L* induces low-level ASFV-specific antibodies in adult breeding boars

**Figure 5:**
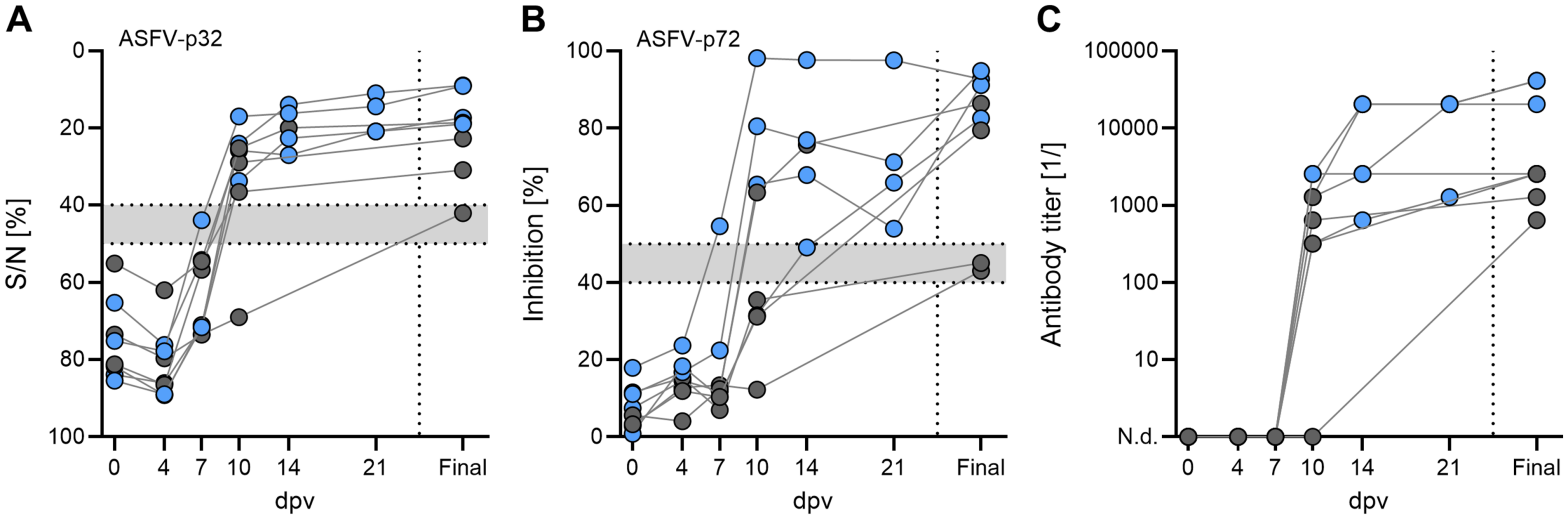
Humoral responses assessed by commercial ELISAs against **(A)** ASFV-p32 and **(B)** ASFV-p72, and **(C)** antibody titers as tested in immunoperoxidase test (IPT) in breeding boars after intramuscular immunization with 1 mL of 10^4.5^ HAU_50_/mL *ASFV-G-ΔI177L*. Animals that succumbed after immunization or reached the HEP are depicted as grey circles. Grey areas between dotted lines in **(A)** and **(B)** indicate assay thresholds, values above were considered positive.

Successful immunization is associated with detection of antigen-specific humoral responses in immunized animals. Therefore, serum samples from all boars were subjected to commercially available ASFV ELISAs [ID Screen African Swine Fever Competition, ID.vet, for anti-ASFV-p32 (Figure 5A) and INgezim PPA Compac, Gold Standard Diagnostics, for anti-ASFV-p72 antibodies (Figure 5B)], and also semi-quantitative titration by immunoperoxidase test (IPT, Figure 5C). Competitive ELISAs showed weakly positive or questionable results for all acutely deceased boars, while all recovered boars developed more pronounced humoral responses. However, all animals were positive for ASFV-specific antibodies as assessed by IPT in endpoint samples, although antibody titers were low in most boars with only two boars reaching titers above 1:10,000.

### 3.5 Immunization with *ASFV-G-ΔI177L* negatively impacts semen quality

**Figure 6:**
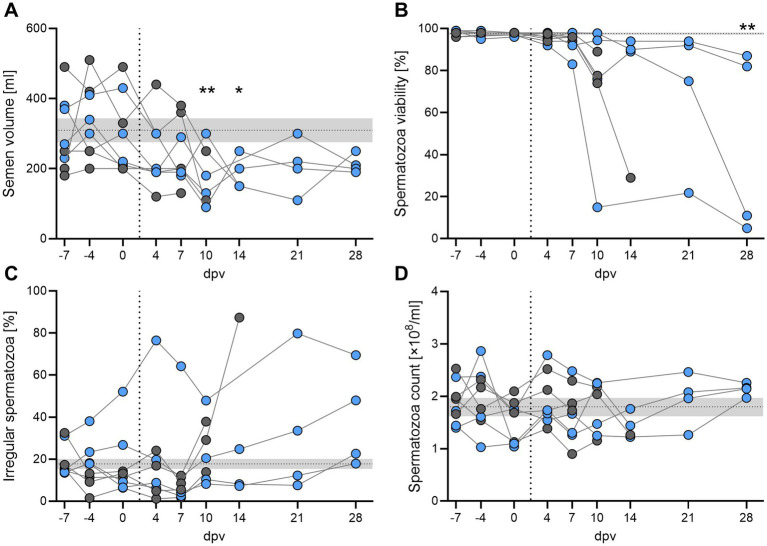
Assessment of semen quality by **(A)** ejaculate volume, **(B)** viability of spermatozoa, **(C)** frequency of morphologically abnormal (irregular) spermatozoa, and **(D)** spermatozoa count per mL ejaculate in breeding boars after intramuscular immunization with 1 mL of 10^4.5^ HAU_50_/mL AS*FV-G-ΔI177L*. Animals that succumbed after immunization or reached the HEP are depicted as grey circles. The horizontal dotted line and grey area indicate mean (SD) of all control samples for each quality marker. The vertical dotted line separates samples taken before and after immunization. Two-way ANOVA with Tukey post-test between grouped pre- and post-immunization samples. *p < 0.05; **p < 0.01.

The effect of *ASFV-G-ΔI177L* on the reproductive performance of active breeding boars was assessed by standard semen quality control assays, i.e., evaluation of ejaculate volume (Figure 6A), spermatozoa viability (Figure 6B), frequency of morphologically abnormal spermatozoa (Figure 6C), and spermatozoa count per mL ejaculate (Figure 6D). To obtain baseline parameters for each individual, semen was collected from all boars thrice prior to immunization (−7, −4, 0 dpv). Beginning 7 dpv, all assessed parameters except for the spermatozoa count began to decline. This decline increased throughout the observation period. Ejaculate volume and spermatozoa viability were still markedly reduced and the frequency of morphologically abnormal spermatozoa was still increased in all surviving boars 28 dpv, despite showing no apparent clinically signs.

### 3.6 Genetic stability of *ASFV-G-ΔI177L*

**Figure 7:**
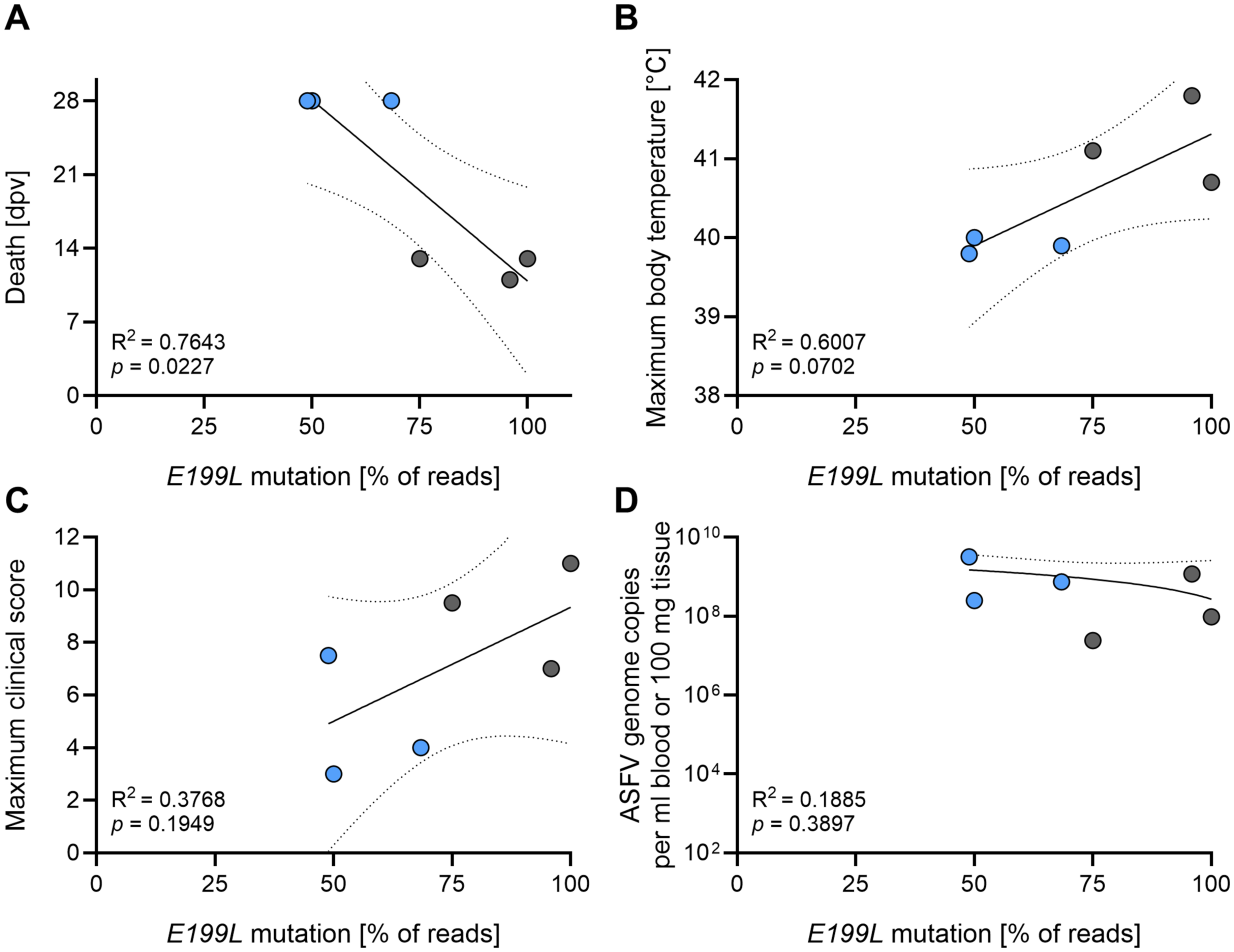
Correlation analysis. Samples from blood and spleen with the highest viral titer were subjected to analysis of genetic stability by sequencing, and correlations were analyzed between the frequency of the E199L mutation and **(A)** time of death **(B)** maximum body temperature, **(C)** maximum clinical score, and **(D)** ASFV genome copy numbers. Two samples with low coverage (boars #2 and #4) were excluded from the analysis. Animals that succumbed after immunization or reached the HEP are depicted as grey circles.

To monitor the genetic stability of the utilized *ASFV-G-ΔI177L* vaccine candidate during the course of the experiment, blood samples of succumbed and surviving animals with the highest available ASFV titer as well as a batch of the administered inoculum were processed for 2nd and 3rd generation sequencing. We found identical genetic changes in the *E199L* gene [position 167.043 in reference to ASFV *Georgia2007* (FR682468.2)], resulting in substitution of serine with phenylalanine (Ser^133^Phe) in the respective ORF (Table 1). These changes were already present in the inoculum (37% of all E199L reads) but increased in frequency, especially in animals that succumbed after immunization (75–100% of all E199L reads) contrary to survivors (48.9–68.4% of all E199L reads). Further analysis revealed a negative correlation of occurrence of the *E199L* mutation with survival (*R*^2^ = 0.7643, *p* = 0.0227, Figure 7A) but positive correlation with fever (*R*^2^ = 0.6007, *p* = 0.0702, Figure 7B). The correlation with clinical signs was less robust, probably due to the HEP as confounder (*R*^2^ = 0.3768, *p* = 0.1949, Figure 7C). This was not caused by an increased viral replication, as the amount of ASFV genome copies did not correlate with occurrence of the *E199L* mutation (*R*^2^ = 0.1885, *p* = 0.3897, Figure 7D).

**Table 1:** Occurrence of an *E199L* mutation in animals immunized with *ASFV-G-ΔI177L*.

Boar ID	Matrix	Time (dpv)	Genome copies/mL or 100 mg	Sequencing platform	Reads total	Reads ASFV	Coverage *E199L*	*E199L* Mutation (%)
1*	Blood	13	9.6E+07	P2 Solo	78,030	98	14	100
2	Blood	7	1.6E+06	P2 Solo	649,627	2	—	—
3	Blood	14	2.5E+08	P2 Solo	1,064,821	139	4	50
4*	Blood	15	7.5E+06	P2 Solo	130,430	89	1	0
5*	Blood	13	2.4E+07	P2 Solo	178,573	110	4	75
6	Blood	14	7.4E+08	P2 Solo	1,071,805	310	19	68.4
7	Blood	14	3.2E+09	P2 Solo	1,084,236	1,567	47	48.9
8*	Blood	11	2.9E+07	P2 Solo	144,892	1,331	49	85.6
8*	Spleen	11	1.17E+09	Illumina	588,319,125	210,988	218	95.9
—	Inoculum	—	—	Illumina	128,439,978	3,925,756	5,986	36.9

*Animals that succumbed after immunization. All E199L mutations were identical [Pos. 167.043 in reference to ASFV *Georgia2007* (FR682468.2)].

## 4 Discussion

With many countries affected and millions of animals lost to the disease itself and along with stringent biosafety measures required to curb outbreaks, the development of a protective and safe vaccine remains an urgent priority. Regulatory authorities mandate comprehensive safety and efficacy studies before licensing a vaccine in the EU. However, many vaccine candidates have failed to meet essential criteria, most often due to insufficient protection from lethal challenge infection, reflecting the still limited understanding of protective immunity to ASFV (Schäfer et al., 2022).

Among the required regulatory evaluations, safety testing in reproductively active animals is a critical requirement. In this study, we investigated the safety of a promising LAV candidate, *ASFV-G-ΔI177L*, which has previously shown to elicit robust protection with a good safety profile in piglets (Borca et al., 2020; Borca et al., 2021; Attreed et al., 2022; Tran et al., 2022a,b; Borca et al., 2023), in adult breeding boars in accordance with regulatory standards (VICH guideline 44). Intramuscular immunization with *ASFV-G-ΔI177L* induced clinical disease in all vaccinated boars, characterized by high fever and associated signs like anorexia and lethargy, especially in those that succumbed later on. Four of the eight vaccinated boars succumbed or reached the HEP after immunization, while the remaining four recovered. Notably, infectious *ASFV-G-ΔI177L* virus was found in semen and tissues of acutely euthanized boars, but persisted in spleens and salivary glands of surviving animals up to 28 dpv, as well as in the semen of two clinically inapparent boars up to 28 dpv.

A similar study has previously shown that boars infected intramuscularly with the naturally attenuated field strain *Estonia14* developed clinically severe ASF and ultimately succumbed after the infection, exhibiting typical pathological findings (Friedrichs et al., 2022; Sehl-Ewert et al., 2023). However, it was still possible to collect semen containing infectious ASFV from clinically inapparent and apparent boars, introducing the potential risk for transmission to sows via artificial insemination. Insemination of sows by standard procedures with semen collected from boars infected with ASFV *Estonia2014* resulted in a 50% infection rate and subsequent loss of most fetuses through reabsorption or abortion (Friedrichs et al., 2022). Analogous transmission pathways have contributed to outbreaks of classical swine fever virus (CSFV) and porcine reproductive and respiratory syndrome virus (PRRSV) (Hennecken et al., 2000; Nathues et al., 2016).

The boars in this study belonged to three different breeds (German Landrace, Large White, Pietrain). While there are indications that factors like genetic background or environment during rearing might have an influence during ASFV infection (Friedrichs et al., 2022; Radulovic et al., 2022; Radulovic et al., 2025), there is no evidence for specific breed-dependent differences in susceptibility. Contrary, it is consensus that all breeds of domestic pigs are equally susceptible to ASFV infection (see, e.g., WOAH Terrestrial Manual 2024, Section 3.9). While the numbers of animals in this study is too small for generalizations, we did not find any robust breed-specific differences. However, the mix of breeds in this study reflects the breeds commonly used in Europe and thus calls for additional studies to assess potential risks of LAV vaccination campaigns.

While the safety profile of *ASFV-G-ΔI177L* has generally been favorable in young pigs (Borca et al., 2020; Borca et al., 2021; Attreed et al., 2022; Tran et al., 2022a,b; Borca et al., 2023), a recent study indicated potential risks for older animals, particularly pregnant sows (van den Born et al., 2025). Here, van den Born et al. (2025) demonstrated residual virulence in pregnant sows immunized in late gestation (100th day of gestation). These results, together with the outcomes in the present study, also indicate that the observed virulence in reproductively active animals is predominately grounded in the animal’s age independent of vaccine dose. While animals in the present study were immunized with 10^4.5^ HAU_50_, sows in the former study were immunized with a hundred-fold smaller dose of 1.5 × 10^2^ TCID_50_, which still caused clinical disease and negatively affected their offspring (van den Born et al., 2025). The analyses by van den Born et al. (2025) also indicated a potential reversion to virulence after forced passages in very young pigs, correlating with an observed mutation in the *C257L* gene. Similarly, the detrimental effects of immunization with *ASFV-G-ΔI177L* in breeding boars observed in our study correlated with a mutation in the *E199L* gene. The *E199L* gene is expressed late after infection and mainly found in viral factories (Sun et al., 1996). Interestingly, the *E199L*-encoded protein has been implicated in a number of viral processes, involving entry, membrane fusion, and core penetration (Matamoros et al., 2020; Cuesta-Geijo et al., 2022), and it has also been shown to induce autophagy, apoptosis, and inflammatory responses in infected cells (Song et al., 2020; Chen et al., 2021; Li et al., 2021; Garcia-Dorival et al., 2023). However, since genetic stability is a crucial safety aspect for LAVs, it needs to be discussed that the mutation in the *E199L* gene was already present in the inoculum, but amplified during the animal passage. It is known that passaging of LAVs on cell lines can already introduce genetic changes, e.g., observed during *in vitro* passaging of LAVs against Influenza (Zhou et al., 2016) and rabies (Borutzki et al., 2022). Additionally, emergence and spread of mutated LAVs might disrupt the success of mass vaccine campaigns, as observed for polio (Kew et al., 2002; Kew et al., 2005; Stern et al., 2017). Therefore, regular genetic screening of vaccine virus batches via deep sequencing is a necessity to facilitate safety in the animal. In our study, an MSV + 2 vaccine (Master Seed Virus) was used, highlighting the importance of early genetic screening. Whether this mutation was causative for the observed clinical severity remains an open question and could be assessed in future studies with the reisolated virus from this trial.

The safety issues in regard to older animals might be relevant in larger pig facilities, where naïve sows or boars could be infected when *ASFV-G-ΔI177L* is shed from younger, vaccinated pigs. This was shown in a previous study, where 50% of contact animals developed ASFV-specific antibodies after comingling with pigs vaccinated intramuscularly with two low doses (10^2.6^ HAU_50_/mL each) of *ASFV-G-ΔI177L* (Tran et al., 2022b). However, we recently emphasized the importance of recognizing that ASFV field infections typically result in 100% lethality, which may justify the use of even an imperfect vaccine in emergency contexts, particularly when implemented into controlled vaccination strategies (Blome et al., 2025). Furthermore, whether safety concerns in reproductively active animals remain if the same individuals were vaccinated as piglets and would therefore not be naïve as adults, remains a crucial open question to address in this context.

Following VICH guideline 44 for vaccine testing, our findings demonstrate that intramuscular immunization of naïve adult boars representative for the global pig breeding landscape with the LAV *ASFV-G-ΔI177L* manufactured to industrial standards can induce considerable clinical disease, result in mortality, and lead to dissemination of infectious virus in several organs, including the reproductive tract. Furthermore, all boars were positive for ASFV genome, some even for infectious viral particles in semen. Additionally, the overall semen quality of all boars decreased significantly, e.g., through reduced viability of spermatozoa and an increase of morphologically abnormal spermatozoa. These outcomes highlight safety concerns for the use of this vaccine in reproductively active boars, with implications for viral transmission via semen and unintended downstream exposure of naïve sows. While the number of animals was relatively small, adherence to internationally recognized standard protocols for vaccine testing ensured that this data set can be used for future licensing purposes. As LAVs remain a promising tool for ASF control, our results aid to establish tailored vaccination strategies, particularly in populations at risk for reproductive consequences.

## Data Availability

The data presented in this study are publicly available. The data can be found at https://www.ebi.ac.uk/ena, accession PRJEB105079.
