# Supplementary material for: Implications for safety: ASFV-G-ΔI177L vaccine compromises health and semen quality in adult breeding boars
Source: Front Microbiol. 2026 Apr 29;17:1823118. doi: 10.3389/fmicb.2026.1823118 (PMC13168198; doi:10.3389/fmicb.2026.1823118)
Supplement: Supplementary file 1 [file Data_Sheet_1.pdf]

**Supplemental Table 1:** General information about the boar in this study. dpv, days post vaccination

| dpv/ Boar # | Age at Immunization [months] | Death [dpv] | Breed |
|---|---|---|---|
| 1 | 12 | 13 | German Landrace |
| 2 | 10 | 28 | German Landrace |
| 3 | 12 | 28 | German Landrace |
| 4 | 17 | 15 | Large White |
| 5 | 13 | 13 | Large White |
| 6 | 9 | 28 | Pietrain |
| 7 | 22 | 28 | Pietrain |
| 8 | 21 | 11 | Pietrain |

**Supplemental Table 2:** Clinical Score in boars after intramuscular immunization with *ASFV-G-ΔI177L* (1ml per individual with $10^{4.5}$ $HAU_{50}$/ml). dpv, days post vaccination

| dpv/ Boar # | -1 | 0 | 1 | 2 | 3 | 4 | 5 | 6 | 7 | 8 | 9 | 10 | 11 | 12 | 13 | 14 | 15 | 16 | 17 | 18 | 19 | 20 | 21 | 22 | 23 | 24 | 25 | 26 | 27 | 28 |
|---|---|---|---|---|---|---|---|---|---|---|---|---|---|---|---|---|---|---|---|---|---|---|---|---|---|---|---|---|---|---|
| 1 | 0 | 0 | 0 | 0 | 0 | 0 | 0 | 0 | 0 | 3 | 2 | 5.5 | 9 | 9.5 | 11 | | | | | | | | | | | | | | | |
| 2 | 0 | 0 | 0 | 0 | 0 | 0 | 0 | 0 | 0 | 0 | 0 | 1 | 2 | 1 | 0 | 0 | 0 | 0 | 0 | 0 | 0 | 0 | 0 | 0 | 0 | 0 | 0 | 0 | 0 | 0 |
| 3 | 0 | 0 | 0 | 0 | 0 | 0 | 0 | 0 | 0 | 3 | 2 | 2.5 | 1.5 | 0 | 1 | 0 | 0.5 | 0.5 | 0 | 0 | 0 | 0 | 0 | 0 | 0 | 0 | 0 | 0 | 0 | 0 |
| 4 | 0 | 0 | 0 | 0 | 0 | 0 | 0 | 0 | 0 | 4 | 4 | 7.5 | 8.5 | 9 | 9 | 9 | 11.5 | | | | | | | | | | | | | |
| 5 | 0 | 0 | 0 | 0 | 0 | 0 | 1 | 3 | 2 | 5 | 4 | 8 | 7.5 | 9.5 | 8.5 | | | | | | | | | | | | | | | |
| 6 | 0 | 0 | 0 | 0 | 1 | 1 | 1 | 1 | 1 | 4 | 3 | 3.5 | 3 | 2 | 3 | 1.5 | 0.5 | 0 | 0 | 0 | 0 | 0 | 0 | 0 | 0 | 0 | 0 | 0 | 0 | 0 |
| 7 | 0 | 0 | 0 | 0 | 0 | 0 | 0 | 2 | 1 | 4 | 4 | 7.5 | 7.5 | 4 | 2.5 | 0 | 0 | 0 | 0 | 0 | 0 | 0 | 0 | 0 | 0 | 0 | 0 | 0 | 0 | 0 |
| 8 | 0 | 0 | 0 | 0 | 0 | 0 | 0 | 0 | 0 | 3 | 3 | 4 | 7 | | | | | | | | | | | | | | | | | |

**Supplemental Table 3:** Rectal Temperature in boars after intramuscular immunization with *ASFV-G-ΔI177L* (1ml per individual with $10^{4.5}$ $HAU_{50}$/ml). dpv, days post vaccination

| dpv/ Boar # | -1 | 0 | 1 | 2 | 3 | 4 | 5 | 6 | 7 | 8 | 9 | 10 | 11 | 12 | 13 | 14 | 15 | 16 | 17 | 18 | 19 | 20 | 21 | 22 | 23 | 24 | 25 | 26 | 27 | 28 |
|---|---|---|---|---|---|---|---|---|---|---|---|---|---|---|---|---|---|---|---|---|---|---|---|---|---|---|---|---|---|---|
| 1 | 38.8 | 37.9 | 38.5 | 38.3 | 38.8 | 37.6 | 38.2 | 38.1 | 38.5 | 38.9 | 40 | 40.1 | 40.7 | 40.7 | 38.8 | | | | | | | | | | | | | | | |
| 2 | 38.6 | 38.6 | 38.8 | 38.4 | 38.5 | 38.3 | 38.5 | 38.5 | 38.2 | 38.7 | 39 | 38.7 | 38.8 | 38.6 | 38.5 | 38.5 | 38.1 | 38.6 | 38.5 | 38.5 | 38.4 | 37.9 | 38.7 | 38.7 | 37.8 | 38.5 | 38.1 | 38.7 | 39.1 | 38.2 |
| 3 | 38.5 | 38.5 | 38.3 | 37.6 | 38.4 | 38.5 | 38.4 | 39 | 39.8 | 39.8 | 39.5 | 40 | 39 | 38.2 | 38 | 39.2 | 38.3 | 38.7 | 38.7 | 38.5 | 38.3 | 38.3 | 38.7 | 38.7 | 38.3 | 39.1 | 38.4 | 38.2 | 38.1 | 39.1 |
| 4 | 38.7 | 38.3 | 38.7 | 38.6 | 38.5 | 38.9 | 38.5 | 39.3 | 39.6 | 40.5 | 40.5 | 40.3 | 40.2 | 40.3 | 40.2 | 39.2 | 39.9 | | | | | | | | | | | | | |
| 5 | 38.5 | 38.5 | 38.8 | 38.2 | 38.2 | 38 | 38.6 | 38.9 | 39.9 | 40.3 | 40 | 40.2 | 41.1 | 40.1 | 39.2 | | | | | | | | | | | | | | | |
| 6 | 38.2 | 38.3 | 37.6 | 37.8 | 38.2 | 38.2 | 38.6 | 39.1 | 39.1 | 39.8 | 39.9 | 39.6 | 38.7 | 38 | 38.3 | 38 | 38.4 | 38.1 | 38.4 | 38.3 | 38.1 | 38.9 | 38.3 | 38.3 | 38.2 | 37.6 | 38.3 | 38.6 | 38.4 | 38.6 |
| 7 | 38.2 | 38 | 37.2 | 37.8 | 37.3 | 37 | 38.8 | 39.8 | 39.4 | 39.7 | 39.8 | 39.3 | 39.7 | 37.1 | 37.6 | 38.7 | 37.2 | 38.5 | 37.8 | 37.8 | 37.7 | 38.4 | 38.1 | 38.1 | 38.4 | 38.3 | 39 | 39.1 | 38.6 | 38.7 |
| 8 | 38.2 | 38.1 | 37.9 | 38 | 37.8 | 37.9 | 38.2 | 38 | 37.8 | 39.6 | 39.8 | 40 | 41.8 | | | | | | | | | | | | | | | | | |

**Supplemental Table 4:** ASFV genome copy numbers in blood, oral fluid, and semen of boars after intramuscular immunization with *ASFV-G-ΔI177L* (1ml per individual with $10^{4.5}$ $HAU_{50}$/ml). dpv, days post vaccination; N.d., not detected

| | dpv/ Boar # | 0 | 4 | 7 | 10 | 14 | 21 | 28 |
|---|---|---|---|---|---|---|---|---|
| **Blood** [per ml] | **1** | N.d. | 1.92E+03 | 7.69E+08 | 5.19E+09 | | | |
| | **2** | N.d. | N.d. | 1.57E+06 | 1.67E+05 | 6.88E+04 | 4.36E+04 | 1.03E+04 |
| | **3** | N.d. | 1.27E+04 | 8.02E+07 | 2.24E+08 | 2.51E+08 | 5.73E+07 | 1.51E+08 |
| | **4** | N.d. | 1.15E+06 | 1.12E+09 | 2.77E+09 | 1.48E+09 | | |
| | **5** | N.d. | 2.37E+05 | 2.48E+09 | 7.00E+09 | | | |
| | **6** | N.d. | 1.88E+04 | 1.05E+08 | 4.23E+08 | 7.39E+08 | 1.30E+08 | 8.39E+07 |
| | **7** | N.d. | 1.56E+06 | 1.61E+09 | 2.38E+09 | 3.16E+09 | 7.32E+08 | No sample |
| | **8** | N.d. | N.d. | 4.72E+08 | 5.63E+09 | | | |
| **Oral Fluid** [per ml] | **1** | N.d. | N.d. | 8.70E+03 | 7.76E+04 | | | |
| | **2** | N.d. | N.d. | 8.94E+03 | 1.97E+05 | 1.33E+06 | 1.05E+05 | 1.76E+05 |
| | **3** | N.d. | N.d. | 6.68E+03 | 1.75E+06 | 5.05E+06 | 8.03E+04 | 1.16E+03 |
| | **4** | N.d. | 1.42E+03 | 8.22E+03 | 6.55E+05 | 8.14E+05 | | |
| | **5** | N.d. | N.d. | 2.40E+04 | 6.98E+05 | | | |
| | **6** | N.d. | N.d. | 2.82E+04 | 2.81E+06 | 1.81E+05 | 2.90E+04 | 4.44E+03 |
| | **7** | N.d. | N.d. | 3.72E+04 | 3.55E+05 | 3.02E+04 | 8.09E+04 | 1.37E+04 |
| | **8** | N.d. | N.d. | 1.44E+05 | 3.38E+05 | | | |
| **Semen** [per ml] | **1** | N.d. | N.d. | 4.35E+03 | No sample | | | |
| | **2** | N.d. | N.d. | N.d. | N.d. | 3.06E+03 | 1.77E+03 | N.d. |
| | **3** | N.d. | N.d. | 2.65E+03 | 2.06E+05 | 1.08E+04 | 3.61E+04 | 1.72E+03 |
| | **4** | N.d. | N.d. | 1.26E+05 | 1.09E+05 | 8.96E+05 | | |
| | **5** | N.d. | N.d. | 1.91E+04 | 1.51E+04 | | | |
| | **6** | N.d. | N.d. | 4.38E+03 | 3.24E+03 | 2.46E+04 | 1.68E+04 | 7.70E+03 |
| | **7** | N.d. | N.d. | 8.57E+02 | 8.66E+05 | 8.13E+07 | 3.19E+07 | 5.62E+05 |
| | **8** | N.d. | N.d. | 1.39E+04 | 3.10E+05 | | | |

**Supplemental Table 5:** ASFV genome copy numbers per 100 mg of the indicated lymphoid and non-lymphoid tissue at the time of necropsy in boars after intramuscular immunization with *ASFV-G-ΔI177L* (1ml per individual with $10^{4.5}$ $HAU_{50}$/ml). N.d., not detected; ghLN, gastro-hepatic lymph node; mLN, mandibular lymph node; gl., gland

| dpv/ Boar # | Tonsil | Spleen | ghLN | mLN | Salivary gl. | Lung | Liver |
|---|---|---|---|---|---|---|---|
| **1** | 6.40E+08 | 1.30E+09 | 1.30E+07 | 5.65E+07 | 7.14E+06 | 6.57E+08 | 5.63E+08 |
| **2** | N.d. | 6.99E+05 | 2.57E+05 | 1.79E+04 | N.d. | N.d. | 6.61E+04 |
| **3** | 6.16E+05 | 1.77E+05 | 2.01E+06 | 3.49E+04 | 3.19E+03 | 3.23E+05 | 8.31E+05 |
| **4** | 3.92E+05 | 3.19E+07 | 2.08E+05 | 9.92E+07 | 2.04E+07 | 8.98E+06 | 1.16E+07 |
| **5** | 1.03E+07 | 5.67E+08 | 1.83E+07 | 4.93E+07 | 1.87E+06 | 3.89E+07 | 6.02E+07 |
| **6** | 2.65E+06 | 1.27E+06 | 2.08E+06 | 6.03E+05 | 4.13E+04 | 6.39E+04 | 9.84E+04 |
| **7** | 7.27E+07 | 4.08E+07 | 4.25E+07 | 2.24E+08 | 9.08E+04 | 6.27E+06 | 1.44E+07 |
| **8** | 8.88E+07 | 1.17E+09 | 9.31E+06 | 2.09E+07 | 1.80E+07 | 1.61E+08 | 1.32E+08 |

**Supplemental Table 6:** ASFV genome copy numbers per 100 mg of the indicated reproductive tissue at the time of necropsy in boars after intramuscular immunization with *ASFV-G-ΔI177L* (1ml per individual with $10^{4.5}$ $HAU_{50}$/ml). N.d., not detected; ghLN, gastro-hepatic lymph node; mLN, mandibular lymph node; gl., gland

| dpv/ Boar # | Testis | Epididymis | Vesiculary gl. | Bulbourethral gl. | Prostate |
|---|---|---|---|---|---|
| **1** | 6.25E+06 | 1.44E+07 | 1.73E+07 | 2.53E+05 | 1.25E+07 |
| **2** | N.d. | N.d. | 9.37E+02 | N.d. | 9.12E+02 |
| **3** | 7.27E+03 | 8.25E+04 | 5.27E+04 | 3.08E+04 | 9.99E+04 |
| **4** | 9.05E+05 | 4.81E+05 | 3.50E+06 | 6.81E+04 | 1.14E+07 |
| **5** | 4.78E+05 | 4.36E+06 | 1.14E+06 | 2.17E+05 | 1.74E+06 |
| **6** | 4.05E+03 | 1.23E+04 | 7.99E+04 | 2.42E+03 | 1.66E+04 |
| **7** | 9.26E+04 | 1.38E+05 | 2.88E+05 | 1.04E+05 | 2.78E+05 |
| **8** | 1.73E+07 | 2.64E+07 | 8.32E+06 | 1.30E+06 | 2.02E+07 |

**Supplemental Table 7:** Assessment of the indicated semen quality parameters of boars after intramuscular immunization with *ASFV-G-ΔI177L* (1ml per individual with $10^{4.5}$ $HAU_{50}$/ml). dpv, days post vaccination

| | **dpv/ Boar #** | **-7** | **-4** | **<u>0</u>** | **4** | **7** | **10** | **14** | **21** | **28** |
|---|---|---|---|---|---|---|---|---|---|---|
| **Ejaculate Volume** [ml] | **1** | 180 | 200 | 200 | 120 | 130 | No sample | | | |
| | **2** | 270 | 340 | 220 | 190 | 190 | 90 | 250 | 200 | 190 |
| | **3** | 370 | 410 | 430 | 300 | 180 | 300 | 150 | 110 | 250 |
| | **4** | 200 | 510 | 330 | 440 | 380 | 250 | 150 | | |
| | **5** | 250 | 250 | 210 | 190 | 200 | 110 | | | |
| | **6** | 230 | 300 | 200 | 200 | 200 | 130 | 200 | 220 | 200 |
| | **7** | 380 | 250 | 300 | 190 | 290 | 180 | Lost | 300 | 210 |
| | **8** | 490 | 420 | 490 | 300 | 360 | 90 | | | |
| **Spermatozoa Viability** [%] | **1** | 98 | 98 | 98 | 97.5 | 96 | No sample | | | |
| | **2** | 99 | 98 | 98 | 98 | 92 | 94.4 | 94 | 94 | 87 |
| | **3** | 99 | 95 | 96 | 98 | 98 | 97.8 | 90 | 92 | 82 |
| | **4** | 96 | 97 | 97 | 94 | 94 | 74 | 29 | | |
| | **5** | 98 | 98 | 98 | 95 | 96 | 77.6 | | | |
| | **6** | 99 | 99 | 98 | 96 | 96 | 76 | 89 | 75 | 11 |
| | **7** | 96 | N/A | 97 | 92 | 83 | 14.9 | Lost | 21.8 | 5 |
| | **8** | 98 | N/A | 98 | 96 | 98 | 89 | | | |
| **Frequency of morphologically abnormal (irregular) Spermatozoa** [%] | **1** | 17.33 | 9.23 | 13.25 | 4.9 | 5.55 | No sample | | | |
| | **2** | 13.81 | 17.8 | 6.6 | 8.76 | 4.29 | 8.19 | 7.22 | 12.24 | 17.91 |
| | **3** | 13.49 | 18.16 | 9.23 | 6.22 | 2.53 | 10.33 | 8.18 | 7.57 | 22.59 |
| | **4** | 32.52 | 13.15 | 14.22 | 24.07 | 8.53 | 29.1 | 87.34 | | |
| | **5** | 14.51 | 11.36 | 11.45 | 16.9 | 12.19 | 37.87 | | | |
| | **6** | 14.86 | 23.38 | 26.81 | 19.69 | 11.54 | 20.52 | 24.84 | 33.54 | 47.97 |
| | **7** | 31.08 | 38.05 | 52.11 | 76.43 | 64.21 | 47.87 | Lost | 79.8 | 69.51 |
| | **8** | 15.45 | 1.52 | 6.39 | 1.16 | 1.78 | 13.88 | | | |
| **Spermatozoa Count per ml Ejaculate** ($\times 10^8$) | **1** | 1.66 | 2.168 | 1.872 | 2.12 | 1.728 | No sample | | | |
| | **2** | 1.44 | 1.03 | 1.096 | 1.736 | 1.304 | 2.248 | 1.44 | 1.96 | 2.144 |
| | **3** | 1.72 | 2.864 | 1.04 | 1.672 | 1.264 | 1.472 | 1.76 | 2.08 | 2.16 |
| | **4** | 1.992 | 2.312 | 1.688 | 1.728 | 1.864 | 2.04 | 1.264 | | |
| | **5** | 2.529 | 1.76 | 2.096 | 2.52 | 2.296 | 2.176 | | | |
| | **6** | 1.4 | 1.608 | 1.76 | 1.544 | 1.664 | 1.248 | 1.224 | 1.264 | 1.968 |
| | **7** | 2.368 | 2.376 | 1.704 | 2.784 | 2.48 | 2.256 | Lost | 2.464 | 2.256 |
| | **8** | 1.948 | 1.544 | 1.128 | 1.384 | 0.9 | 1.152 | | | |

**Supplemental Table 8:** Humoral responses assessed by commercial ELISAs against ASFV-p32 (% S/N) and ASFV-p72 (% Inhibition), and antibody titers as tested in immunoperoxidase test (1/x) in boars after intramuscular immunization with *ASFV-G-ΔI177L* (1ml per individual with $10^{4.5}$ $HAU_{50}$/ml). dpv, days post vaccination

| | dpv/ Boar # | 0 | 4 | 7 | 10 | 14 | 21 | Final |
|---|---|---|---|---|---|---|---|---|
| **ASFV-p32** (negative: >50%, questionable: 50-40%, positive: <40%) | 1 | 55.07 | 62.00 | 54.50 | 36.51 | | | 30.91 |
| | 2 | 85.46 | 88.93 | 71.58 | 33.71 | 22.59 | 20.88 | 18.98 |
| | 3 | 75.10 | 77.81 | 43.83 | 16.99 | 16.18 | 14.37 | 9.04 |
| | 4 | 81.22 | 86.49 | 56.67 | 25.21 | 19.97 | | 18.62 |
| | 5 | 81.79 | 89.20 | 71.03 | 28.92 | | | 22.68 |
| | 6 | 65.25 | 76.19 | 54.13 | 25.76 | 27.02 | 20.88 | 17.35 |
| | 7 | 83.90 | 86.04 | 71.49 | 24.04 | 13.92 | 10.94 | 8.95 |
| | 8 | 73.47 | 79.62 | 73.47 | 68.96 | | | 42.02 |
| **ASFV-p72** (negative: <40%, questionable: 40-50%, positive: >50%) | 1 | 3.30 | 11.90 | 10.35 | 31.19 | | | 79.45 |
| | 2 | 0.84 | 18.33 | 10.35 | 31.49 | 49.08 | 65.85 | 82.56 |
| | 3 | 11.14 | 16.78 | 22.39 | 80.49 | 76.87 | 71.18 | 94.83 |
| | 4 | 5.64 | 4.07 | 12.42 | 63.34 | 75.68 | | 86.33 |
| | 5 | 11.53 | 15.30 | 6.95 | 35.48 | | | 45.08 |
| | 6 | 7.44 | 14.56 | 11.90 | 65.41 | 67.85 | 53.95 | 91.13 |
| | 7 | 17.91 | 23.65 | 54.62 | 98.15 | 97.63 | 97.56 | 92.68 |
| | 8 | 2.86 | 12.79 | 13.38 | 12.27 | | | 43.02 |
| **IPT Titer** | 1 | neg | neg | neg | 320 | | | 2560 |
| | 2 | neg | neg | neg | 320 | 640 | 1280 | 2560 |
| | 3 | neg | neg | neg | 2560 | 2560 | 20480 | 20480 |
| | 4 | neg | neg | neg | 1280 | 2560 | | 2560 |
| | 5 | neg | neg | neg | 640 | | | 1280 |
| | 6 | neg | neg | neg | 1280 | 20480 | 20480 | 40960 |
| | 7 | neg | neg | neg | 2560 | 20480 | 20480 | 40960 |
| | 8 | neg | neg | neg | neg | | | 640 |
